# TMJ Dysfunction and Systemic Correlation

**DOI:** 10.3390/jfmk5010020

**Published:** 2020-03-09

**Authors:** Luca Fiorillo, Giuseppe Musumeci

**Affiliations:** 1 Department of Biomedical and Dental Sciences, Morphological and Functional Images, University of Messina, 98100 Messina ME, Italy; 2 Department of Biomedical and Biotechnological Sciences, Anatomy, Histology and Movement Sciences Section, School of Medicine, University of Catania, Via Santa Sofia 87, 95123 Catania, Italy; g.musumeci@unict.it; 3 Research Center on Motor Activities (CRAM), University of Catania, Via Santa Sofia 97, 95123 Catania, Italy; 4 Department of Biology, Sbarro Institute for Cancer Research and Molecular Medicine, College of Science and Technology, Temple University, Philadelphia, PA 19122, USA

**Keywords:** TMJ, dentistry, posturology, systemic disease, syndrome, occlusion, skeletal muscle

## Abstract

In recent years it has been conclusively shown how the position of the mouth in relation to the body affects the way of walking and standing. In particular, occlusion, the relationship between skull and jaw, swallowing and convergence of the eyes are in neuro-muscular relationship with the control and maintenance system of posture, integrating at different levels. This manuscript aims to be a summary of all the oral, occlusal and articular dysfunctions of TMJ with systemic and postural–muscular repercussions. Recent articles found in the literature that are taken into consideration and briefly analyzed represent an important starting point for these correlations, which are still unclear in the medical field. Posturology, occlusal and oral influences on posture, spine and muscular system are still much debated today. In the literature, there are articles concerning sports performance and dental occlusion or even the postural characteristics of adolescents or children in deciduous and mixed dentition. The temporomandibular joint, as the only joint of the skull, could therefore represent a site to pay particular attention to, and in some cases an ATM dysfunction could be a clue for the diagnosis of systemic pathologies, or it could be the repercussion.

The term posture refers to the strategy used by the neuromuscular and skeletal system to remain in balance, reacting to the force of gravity. In fact, the stomatognathic system actively participates in maintaining the correct position of the skull together with the temporo-mandibular joint (TMJ). In the diagnostic field, important help is offered by dental kinesiology, which allows us to distinguish and analyze the different postural biomechanical problems. Neurophysiologists are unable to explain exactly how the body posture is actually regulated from the neuromuscular point of view. However, it should be considered that the “postural system” is integrated by information that comes from the visual system, from the vestibular system and from the proprioception system, which comes mainly from the musculature of the mechano-receptors of the neck and even the feet. The human body orients in space by recognizing forceful factors, such as gravity, and modes of support, such as the ground to support the body while upright or a chair to support the body while seated, within the world around us. Stabilization is the process of stiffening one part of the body to allow free movement of another body part within the normal range of motion. An essential scheme of the mechanism just described is shown in Figure 1. Posturo-graphic investigations are said not to have consistently found a relationship between occlusion and body posture. This result is due to the numerous compensation mechanisms that intervene in the maintenance of homeostasis by the neuromuscular system that the instrumentation cannot evaluate. In addition, research shows that temporomandibular disorders (TMD) are not always linked to specific occlusal problems and have no documented relationship with head or body posture. According to this study, there is no evidence between occlusal and postural characteristics, and it is clear that the presence of pain in the TMJ is not connected to the existence of a demonstrable occluded-postural anomaly, therefore, the use of tools and techniques for measuring presumed occlusal abnormalities (electromyography, kinesiography, posturography, etc.) cannot be justified in evidence-based medicine [1,2,3,4,5,6].

Stomatognathic systems perform different roles, such as in breathing, as can be summarized with some concepts. Breathing is the first function to appear and the last to disappear; the cranio-maxillofacial complex has close links with the entire rib cage (visceral chain anteriorly and vertebral posteriorly); all these structures have a common membranous origin, and therefore there is a link in the harmonic or pathological development. The postural system is a complex system that cannot be reduced to the position of some teeth. The usual physiological occlusion occurs when there is a correct relationship between the maxillary bone and the jaw, where the dental contact should always be uniform and simultaneous, in order to give the jaw maximum stability with an adequate number of contacts, with tone muscles and asymptomatic temporomandibular joints (Figure 2) [7,8].

When an alteration of chewing occurs, for example a dental precontact (one cusp of the tooth that touches before the others at the time of dental closure), there could be defined a pathological occlusion, and in other words of a "new occlusion", which determines a new mandibular position (dislocation) and therefore a new posture. It is interesting to understand the sequence of events that occur when the situation just described occurs and that lead to changes in the postural structure. The precontact or occlusal interference determines an immediate reflex which is called an "avoidance reflex" and which can also occur in other circumstances [9,10,11].

The first diagnostic step is a postural and gnathological examination to be able to correlate the disorders reported by the patient and their actual cause. An important step is the "deprogramming of chewing" and the analysis of occlusal and temporomandibular joint disorders, that is, visualization of the cause of bad chewing, immediate verification of normalization of muscle tone and change of posture. Diagnostic–investigative help could be given by the stabilometric platform (tool that highlights postural asymmetries) and by the data (tool that measures the support and load of the foot). All these steps are confirmed by the responses to dedicated kinesiological tests. After carrying out this first analysis, if there are dysfunctions starting from the eyes or from the tongue, an orthoptic or speech therapy examination is carried out. Short-term therapy with a personalized and balanced bite to correct problems of dental origin and test postural normalization. This short course of a few months could be accompanied by physiotherapy and osteopathic, orthoptic and speech therapy cycles. This short therapy with the bite leads to the resolution of contracture and painful problems in the neck and back, with consequent wellbeing and regained energy. Once the wellbeing is confirmed, a re-evaluation is carried out to define a definitive dental therapy, replacing the bite, which leads to the maintenance of the new wellbeing acquired [12,13,14,15,16,17,18,19,20,21].

There is an important correlation between sports activity and occlusal activity, as during periods of intense activity or particular concentration it is possible that occlusal parafunctions occur. Certain orthodontic appliances such as bites may in some cases assist in releasing these occlusal forces [22]. It is important to consider how alterations in the temporomandibular joint can also represent a first step towards an early diagnosis of some pathologies, such as in the case of fibromyalgia [23,24,25,26]. Evaluating instrumental methods [27] for an early diagnosis or for a quick resolution of joint problems can be a good starting point for improving the quality of life related to oral health (OHRQoL) [28,29,30].

## Figures and Tables

**Figure 1:**
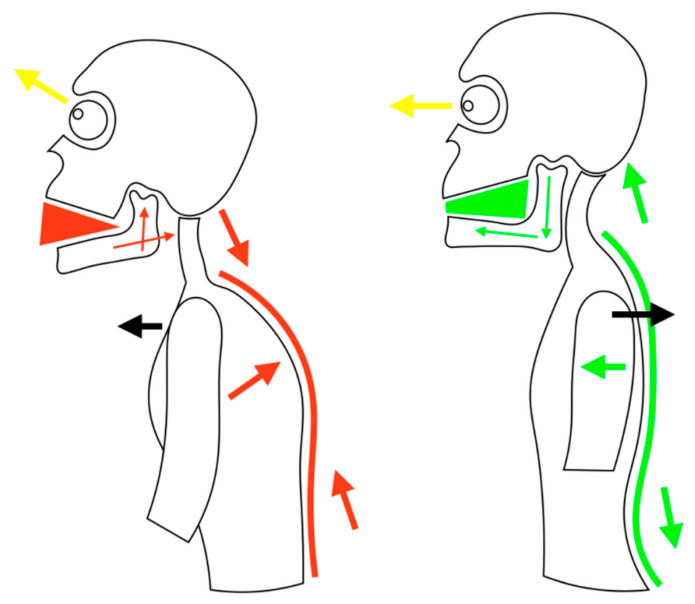
TMJ, posture and airways. Note how mandible position is strictly related with trunk posture. https://www.mdpi.com/2411-5142/4/3/58. By gentle concession of S. Sambataro [3].

**Figure 2:**
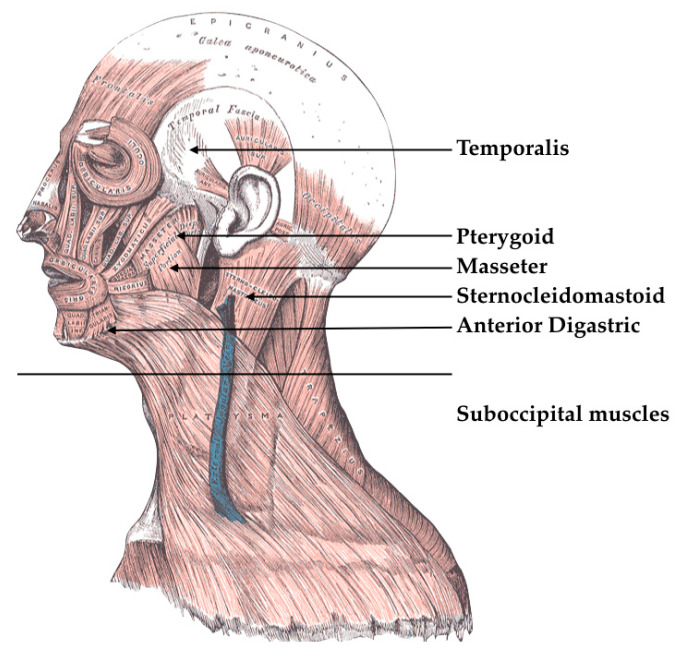
Masticatory muscles. https://www.mdpi.com/2411-5142/5/1/7. By gentle concession of R. De Stefano.
